# Supplementation of urea to a basal pasture diet fed to dairy cows to model N-partitioning relationships

**DOI:** 10.3168/jdsc.2020-0016

**Published:** 2020-12-11

**Authors:** S.J. Hendriks, N. Lopez-Villalobos, A.J. Sheahan, D.J. Donaghy, J.R. Roche

**Affiliations:** 1 School of Agriculture and Environment, Massey University, Palmerston North 4442, New Zealand; 2 DairyNZ Ltd., Hamilton 3240, New Zealand; 3 School of Biological Sciences, University of Auckland, Private Bag 92019, Auckland 1142, New Zealand

## Abstract

The main objective of this study was to investigate whether altering dietary crude protein (CP) through the supplementation of urea to a basal pasture diet fed to dairy cows accurately modeled N-partitioning relationships. To test this, we first needed to establish safe tolerance levels for urea in this setting. Fifteen multiparous, rumen-fistulated, mid-lactation Holstein-Friesian dairy cows were offered spring pasture (~20 kg of dry matter/cow per day) and allocated to 1 of 3 urea supplementation treatments: low N [0 g/d urea; 21% total dietary CP of dry matter (DM)], medium N (350 g/d urea; 26% total dietary CP of DM), or high N (690 g/d urea; 31% total dietary CP of DM), in a completely randomized design. The amount of urea provided daily increased gradually for all cows over a 21-d period, with target urea supplementation reached by d 21. Milk yield decreased linearly at a rate of 2.35 kg/100 g of urea intake when urea supplementation exceeded 350 g/d for 4 d (~2% of DM intake). Cows from the low- and medium-N treatments subsequently entered metabolism stalls from d 25 to 31 to collect urine, feces, and milk for total N collection. Estimated urinary N output (g/d) increased linearly with N intake (g/d), and the slope of the relationship (slope = 0.86; R^2^ = 0.82) was consistent with international published results. Because of the consistency of our results with previously documented relationships, our findings indicate that supplementation of urea to a basal pasture diet is a suitable technique for modeling different N intakes from pasture diets to evaluate urinary N mitigation strategies. Urea supplementation, however, should not exceed ~2% of DM intake.

## Highlights

• Urinary nitrogen from grazing cows is a major source of nitrogen losses to waterways
• Nitrogen balance studies can be used to evaluate nitrogen mitigation strategies
• We modeled nitrogen partitioning relationships using a novel technique
• Supplementing urea to a pasture diet could be used to test mitigation strategies

## Graphical Abstract

**Figure:**
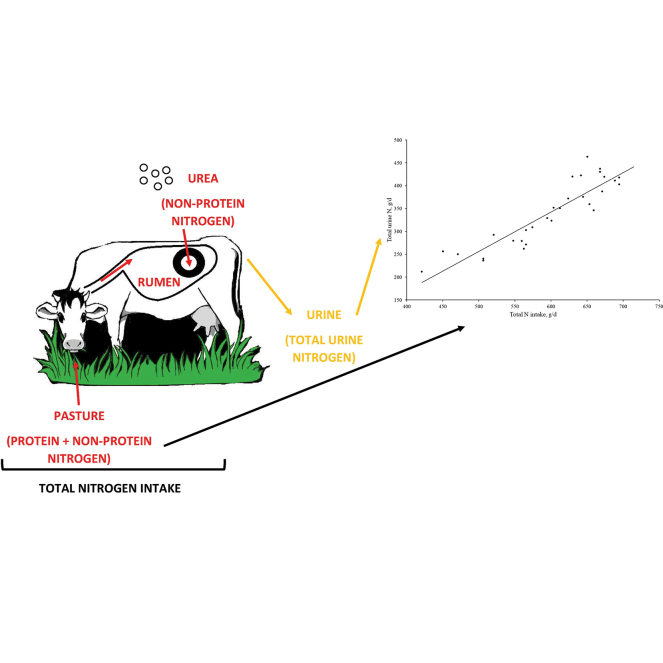
**Summary:** Nitrogen balance studies using pasture-fed cows may be useful in trialing strategies for reducing urinary nitrogen deposition on pasture. Being able to alter dietary nitrogen is critical to being able to evaluate mitigation strategies, but it is difficult to investigate differing levels of dietary nitrogen in dairy cows fed pasture, without confounding other nutritional variables. This study investigated the use of urea supplementation in rumen-fistulated dairy cows as an experimental model of varying dietary nitrogen content for cows fed pasture without altering other dietary components. Relationships between both nitrogen intake and urinary nitrogen output were consistent with those reported in international studies. Urea supplementation could be used as a novel experimental model to evaluate urinary nitrogen mitigation strategies.

Perennial ryegrass (*Lolium perenne* L.) is the most widely used grass species in temperate, pasture-based production systems (Kemp et al., 2002). It is a highly digestible forage, with ME concentrations and milk production per kilogram of DMI similar to that of corn grain (Roche et al., 2010; Macdonald et al., 2017); however, it has high requirements for N (Whitehead, 1995), which results in concentrations of CP and, in particular, RDP that are in excess of dairy cow requirements (Roche et al., 2009, 2016). There is little scope for reducing perennial ryegrass N requirements through conventional plant breeding because there is very little intra-species variation (Chapman et al., 2015). These high CP and RDP concentrations in pasture can be further exacerbated by short regrowth intervals between grazings and by N fertilizer inputs (Martin et al., 2017).

Excess consumption of CP by grazing dairy cows can result in high concentrations of N being deposited on pasture in urine patches (Haynes and Williams, 1993). Nitrogen surplus to pasture requirements for growth is susceptible to leaching into surface and groundwater (Haynes and Williams, 1993). Urinary N losses from farm animals are, therefore, the major source of N in waterways draining agricultural catchments in which animals predominantly graze pasture (Scarsbrook and Melland, 2015). Strategies that reduce the amount of N leaching from pastoral farming systems are a key sustainability priority; because the urine patch of dairy cows is a significant contributor to N leaching, many of the mitigation strategies aim to reduce the amount and concentration of urinary N that is deposited onto pasture at the animal level (de Klein et al., 2010).

An experimental model that allows the manipulation of dietary CP concentrations without altering other nutrients involved in N partitioning in the animal is required to investigate the effects of potential mitigation strategies aimed at reducing urinary N output (e.g., cow genetics). Unlike TMR systems, however, where the ration can be formulated to provide dietary treatments with different CP concentrations while maintaining similar concentrations of structural and NSC concentrations, pasture systems have many interacting factors that influence the CP concentration of the pasture ingested. Crude protein concentrations vary due to climate, time of day, species, sward composition, regrowth interval, and N fertilizer application (van Vuuren et al., 1991; Roche et al., 2009; Keim and Anrique, 2011). These factors also affect the concentration of dietary NSC, which can also affect N partitioning within the animal, undermining the applicability of the research (van Vuuren et al., 1991).

In experimental work with cows offered TMR, diets differing in N content have been created by offering a basal diet to all subjects and supplementing with urea to allow the levels of N in the diet to be increased while maintaining similar concentrations of other nutrients (Burgos et al., 2007). To our knowledge, there is limited information of this approach being attempted in cows fed a basal pasture diet. The base pasture diet is already high in CP, however, and consuming high levels of dietary NPN has reportedly reduced DMI and milk production, at least in housed cows consuming a TMR (Van Horn et al., 1967; Kertz et al., 1982). Because low CP is rarely, if ever, a production limitation in cows consuming predominantly fresh pasture, there is little information regarding the implications of using urea to increase dietary N intake in this scenario. Therefore, our first objective was to establish safe tolerance levels for urea supplementation in dairy cows fed a basal diet of fresh temperate pasture.

Although we acknowledge that the nutritional value of urea is not the same as pasture total protein, we hypothesized that supplementing fresh pasture with urea could be used as a model to increase dietary N concentrations in cows consuming fresh pasture while maintaining the concentrations of all other nutrients. Our second objective, therefore, was to investigate whether altering CP concentrations through the supplementation of urea to a basal pasture diet fed to dairy cows resulted in N-partitioning relationships similar to previously published equations from international studies.

This study was conducted in 2 phases at the DairyNZ Lye Farm, Hamilton, New Zealand (37°46′ S, 175°18′ E). Phase 1 was designed to establish safe tolerance levels for urea supplementation, and phase 2 investigated partitioning of N between feces, urine, and milk in dairy cows with stable BW (103 DIM). The Ruakura Animal Ethics Committee (Hamilton, New Zealand) approved all animal manipulations.

In phase 1, 15 multiparous, rumen-fistulated, mid-lactation Holstein-Friesian cows (mean ± standard deviation, **SD**; 103 ± 9.4 DIM; 505 ± 10.4 kg of BW; 4.1 ± 0.04 BCS) were allocated to 1 of 3 urea supplementation treatments (n = 5 cows/treatment): low N (0 g/d urea; 21% total dietary CP of DM), medium N (351 g/d urea; 25% total dietary CP of DM; supplementary NPN from urea 20% CP), and high N (690 g/d urea; 31% total dietary CP of DM; supplementary NPN from urea 32% CP), in a completely randomized design. This was equivalent to 0 and, approximately, 2 and 4% of cow DMI, respectively. The number of animals used was derived from a power analysis based on standard deviations from prior metabolism stall experiments. Cows had access to a fresh allocation of pasture twice daily after the a.m. and p.m. milkings; they were offered 20 kg (DM)/d of high-quality pasture throughout phase 1. Nitrogen fertilizer was not applied to the swards used in the experiment for at least 6 wk before the experiment to provide a relatively low CP concentration in fresh pasture (21% DM).

The Animal Ethics committee required that cows were gradually acclimated to their urea treatment over 21 d in phase 1 (October 12 to November 1, 2009) to minimize the risk of urea toxicity. Urea was introduced through the rumen fistula in the paddock (i.e., grazing area) approximately 30 min after pasture allocation (i.e., 0930 and 1630 h) to coincide with the rapid degradation of RDP in pasture. When the daily dosage amount was ≥351 g of urea/d, the urea was provided in 3 doses with an additional dose at 1300 h. All cows had ad libitum access to fresh water.

The gradual introduction allowed us to identify the maximum safe level of urea supplementation for cows grazing fresh temperate pasture (i.e., defined as the urea level above which DMI and milk production were negatively affected). The acclimation routine was as follows:

• cows in both medium-N and high-N treatments received 44 g 2×/d (i.e., 88 g/d) for 4 d;
• cows in both medium-N and high-N treatments received 88 g 2×/d (i.e., 176 g/d) for 3 d;
• cows in both medium-N and high-N treatments received 117 g 3×/d (i.e., 351 g/d) for 4 d;
• cows in the high-N treatment received 150 g 3×/d (i.e., 450 g/d) for 3 d;
• cows in the high-N treatment received 200 g 3×/d (i.e., 600 g/d) for 4 d; and
• cows in the high-N treatment received 230 g 3×/d (i.e., 690 g/d) for 3 d.

The literature is limited in its information regarding urea supplementation to grazing cows; therefore, to be safe, our urea dose rates were within ±1% of rates (% of DMI) reported in the literature (Van Horn et al., 1967). To ensure that cow health and welfare was not compromised, cows were visually observed for signs of ammonia toxicity during the acclimation period (i.e., lethargy, grazing behavioral changes). To avoid any potential confounding effects of different lengths of time on the final urea dose, the medium-N treatment group began receiving their urea supplementation on d 11 of urea supplementation for the high-N treatment. In that way, both medium- and high-N treatments reached their final urea dose on the same day.

Milk yield during phase 1 declined at a rate of 2.35 kg/100 g of urea intake when urea intake increased beyond 350 g/d, for 4 d (approximately 2% of DMI; Figure 1; *P* < 0.001). This agrees with Van Horn et al. (1967), who reported that the inclusion of 2.2 and 2.7% urea in a TMR diet reduced milk yield. Dry matter intake decreases when urea's contribution to total dietary CP exceeds 30% (Polan et al., 1976); thus, if the administration of urea decreases DMI, energy intake and the relative efficiency of energy utilization for milk yield are decreased (Van Horn et al., 1967; Kertz et al., 1982). Although individual DMI could not be directly measured while cows were grazing, back calculations from milk yield data support the premise that DMI was reduced in cows as urea supplementation exceeded 25% of dietary CP concentration. These results indicate that when urea is used to create high-N diets, the urea supplemented should not exceed ~2% DMI or 25% of dietary CP.

**Figure 1:**
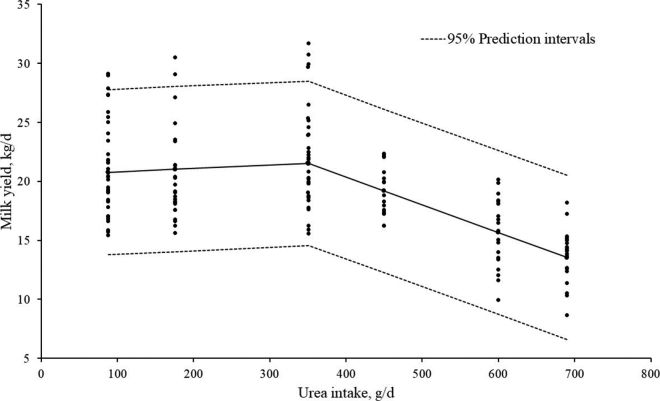
Relationship between urea intake (g/d) and milk yield (kg/d) for all cows during phase 1. If *x* ≤350 g of urea/d, then *y* = 20.5 + 0.0029*x*; if *x* >350 g of urea/d, then *y* = 20.5 + {350 × [0.0029 – (–0.0235)]} + (–0.0235*x*); *P* < 0.001.

In response to the safe tolerance levels established in phase 1, urea supplementation in the medium-N treatment was reduced from 350 to 250 g/d, equivalent to 1.25% of DMI, to ensure that animals received a safe dose of urea during phase 2, when the N-balance study was undertaken. On d 22, cows from the low-N and medium-N treatments entered the metabolism stalls, and total urine, feces, and milk were collected from d 25 to 31. While housed in metabolism stalls, cows from the low-N and medium-N treatments were offered 20 kg (DM) of pasture (~100 kg wet weight of fresh pasture) daily. Pasture was mown and collected in a single chop with no further processing postharvest (~150-mm chopped length). The daily allowance was split, and ~11 kg DM/cow was offered at 0900 h, with the remaining 9 kg DM/cow offered at 1600 h following milking.

During phase 1, when cows were grazing outdoors, representative samples of pasture (~500 g of fresh pasture) were collected on 3 d each week by plucking pasture to grazing height from the day's pasture allocation due to be grazed, and samples were bulked weekly. When cows were in the metabolism stalls (phase 2), pre- and postfeeding pasture samples were collected daily from individual cows and bulked weekly. During phase 1, all samples were analyzed for CP, NDF, and ADF; during both phase 1 and 2, samples were analyzed for NSC, DM digestibility, and ME by near-infrared spectroscopy (Feed Tech, Palmerston North, New Zealand), as described by Corson et al. (1999). During phase 2, all samples were analyzed for CP, NDF, and ADF by wet chemistry (Dairy One Analytical Services, New York, NY). The pasture offered during phases 1 and 2 had a mean (±SD) ME of 11.8 ± 0.06 and 12.0 ± 0.13 MJ/kg of DM, respectively. Mean ± SD for CP, NDF, ADF, NSC, and DM digestibility was 20.7 ± 0.56, 39.5 ± 0.52, 21.8 ± 0.36, 19.6 ± 0.69, and 83.3 ± 0.45% DM during phase 1, respectively. Further, during phase 2, mean ± SD for CP, NDF, ADF, NSC, and DM digestibility were 18.4 ± 1.69, 43.1 ± 1.10, 25.3 ± 0.89, 22.7 ± 2.41, and 79.0 ± 0.72% DM, respectively.

Individual cow milk yield was recorded daily using Westfalia Surge (GEA, Hamilton, New Zealand) during phase 1 and Tru-Test milk meters (Palmerston North, New Zealand) during phase 2. Milk composition was determined on composite afternoon and morning samples collected weekly during phase 1 and daily during phase 2 using a Fossomatic FT120 (Foss Electric, Hillerød, Denmark). In phase 2, milk N % was calculated by dividing milk CP % by 6.38. Feces were collected using urine and fecal separators and weighed daily. A fecal sample was collected at 0900 h daily from the bulk feces collected during the day and analyzed to determine fecal N concentration. Additional fresh duplicate samples (~200 g of wet weight per sample) were collected daily and analyzed for composite DM content (DairyNZ Analytical Services, DairyNZ Ltd., Hamilton, New Zealand). Measured fecal N content (N %) and dry fecal weight were used to calculate the total fecal N excreted (g/d).

Total DM and N intake (from pasture plus urea) were used to calculate the CP content of the diet (% DM: CP = N × 6.25; NRC, 2001). During phase 1, DMI was estimated by back-calculating ME requirements of the cows, as recommended by Nicol and Brookes (2007), using the BW of each cow (maintenance = 0.56 MJ/kg of BW^0.75^), activity (0.0037 MJ/kg of BW per horizontal kilometer walked), BW change (regressed over time to predict the average daily change), milk yield and composition [milk energy = (0.0929 × fat % + 0.0547 × CP % + 0.0395 × lactose % × milk yield)/0.65 × 0.238 Mcal/MJ; Holmes et al., 1981], and dividing by the average ME content of the pasture consumed. During phase 2, DMI was calculated from pasture offered less pasture refused in the metabolism stalls. Nitrogen balance was calculated using measured N intake, fecal N, and milk N (assuming that N retention was negligible). Although urine volume was measured, N concentrations could be underestimated due to losses of volatile NH_3_ from the urine collection containers. The addition of large volumes of acid to the urine collection containers to lower the pH and minimize N losses (Knowlton et al., 2010) was deemed a health and safety risk by the DairyNZ Health and Safety Committee. Instead, we estimated urinary N (g/d) as the difference between total N intake and the sum of total fecal and milk N, as described by Spek et al. (2013); we acknowledge that this method is not ideal, because it assumes N retention is zero. Nevertheless, we are confident that the retained N was negligible as cows were not pregnant and in mid-lactation, when BCS change is near zero and protein accretion in the gravid uterus and lean tissue are negligible (Bell et al., 1995; Roche et al., 2007). Yet, we acknowledge that this and the estimation of urine N yield are limitations of our approach.

Statistical analyses were undertaken using SAS 9.3 software (SAS Institute Inc., Cary, NC). Phase 1 analyses included data from 15 cows across the 3 treatment groups. The relationship between urea intake (independent variable) and milk yield (dependent variable) was examined by piecewise regression analysis (PROC NLIN) to determine the point of inflection where the most significant change in milk yield occurred relative to urea intake. In phase 2, data from 10 cows across the low- and medium-N treatment groups were available and included in subsequent analyses. The difference between the 2 treatment groups for nitrogen balance, milk yield, and components were analyzed using a repeated-measures ANOVA (PROC MIXED). The model included the fixed effect of treatment and the random effect of cow, and least squares means and standard errors of the mean are presented. Variables were checked for skewness and to meet the assumption of normal distribution. A linear regression equation was estimated for the relationships between total urinary N and N intake for all cows using the GLM procedure.

Nitrogen parameters pertaining to N partitioning between feces, milk, and urine in phase 2 are presented in Table 1. Urea supplementation increased estimated urinary N concentration (*P* < 0.05) and daily output of N in urine (*P* < 0.001; Table 1); furthermore, the increase in estimated urinary N output with N intake/day was linear (*P* < 0.001; Figure 2). Urea supplementation of dairy cows consuming adequate CP from spring pasture (18% CP) increased fecal N concentration (*P* < 0.05), but the daily output of N in either feces or milk did not differ between treatments. A positive effect of N intake on fecal and milk N output has been previously reported (Castillo et al., 2000; Kebreab et al., 2001); however, these studies formulated high-CP diets using TMR. We cannot determine with certainty why fecal and milk N output were not affected, but it is possible that the experimental model we used resulted in rapid conversion of additional dietary N to NH_3_ in the rumen, which would have been quickly absorbed. Therefore, no improvement in MP outflow from the rumen was expected (Macdonald et al., 1998; Kebreab et al., 2001), and no change in either fecal N output or milk protein production occurred (Castillo et al., 2000). During the total N collection period (phase 2), milk yield was not different (*P* = 0.33) between the low-N and medium-N treatments (25.0 and 22.9 kg/d, respectively). Further, milk CP and fat percentage were not different (*P* = 0.34 and 0.81, respectively) between the low-N (3.33 and 4.23%, respectively) and medium-N treatments (3.55 and 4.32%, respectively). It must be acknowledged, however, that statistical power to detect a difference in milk production was, admittedly, limited by low replicate numbers.

**Table 1:** Mean nitrogen parameters pertaining to N partitioning in urine, feces, and milk from mid-lactation cows consuming freshly cut pasture (18% CP of DM; low N) and pasture plus 250 g of urea/cow per day (23% CP of DM; medium N) during the total N collection period (phase 2)

Parameter^1^	Low N	Medium N	SEM	*P*-value
CP concentration, % of DM	18.4	22.6	0.08	<0.001
N intake, g/d	563	674	17.2	<0.001
Urine N, g/L	8.7	11.7	0.67	<0.05
Total estimated urine N,^1^ g/d	309	422	14.2	<0.001
Fecal N, g/kg	3.04	3.12	0.04	0.05
Total fecal N, g/d	133	132	9.91	0.91
Milk N, %	0.52	0.56	0.03	0.34
Total milk N, g/d	123	126	5.31	0.62

1 Total estimated urine N (g/d) = total N intake – (total fecal N + total milk N). It was assumed that N retention was negligible (Spek et al., 2013).

**Figure 2:**
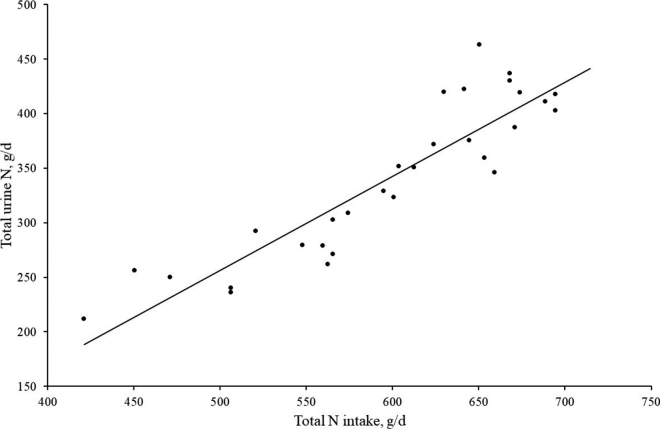
Relationship between total estimated urine nitrogen (N; g/d) and total N intake (g/d) for the low-N and medium-N cows during the total N collection period (phase 2). Estimated daily urine N output = −174.2 + 0.86 × total N intake; R^2^ = 0.82. Each black dot represents data for an individual cow on a single day during the total N collection period.

Our results indicate that N intake was a strong predictor of estimated urinary N output (*P* < 0.001; –174.2 + 0.86 × total N intake; R^2^ = 0.82); however, the difficulty in measuring dietary N intake in pasture-based systems is a major limitation of N intake as a predictive measure for urinary N. Only when reliable measures of dietary DMI—and thus dietary N intake—in grazing cows become available can urinary N output be predicted using N intake. Nevertheless, a positive relationship between N intake and urinary N output has been established previously in housed cows fed TMR (Castillo et al., 2000; Kebreab et al., 2001; Marini and Van Amburgh 2003; Colmenero and Broderick, 2006) and in one study in dairy cows grazing pasture (Moorby, 2014). Consistent with Marini and Van Amburgh (2003) and Colmenero and Broderick (2006), our results indicated a linear increase in urinary N output with increasing N intake. In contrast, however, Castillo et al. (2000) and Kebreab et al. (2001) reported a quadratic relationship between N intake and urinary N output, and Moorby (2014) reported a split-line relationship, with a point of inflection around 400 g of N intake/d. The reason for the inconsistency in reported results probably relates to differences in base N intakes across experiments. For example, in this study, N intake ranged from 420 to 750 g of N/d; therefore, our data points are above the point of inflection described by Castillo et al. (2000), Kebreab et al. (2001), and Moorby (2014). In the current study, when N intakes ranged from 400 to 600 g of N/d, between 170 and 342 g of N/d was excreted in urine. This is remarkably similar to the 124 to 300 g of N/d excreted in urine for the same N intakes reported by Castillo et al. (2000), Kebreab et al. (2001), and Moorby (2014), despite considerable differences in experimental models, cow genetics, daily milk protein yield, and sources of N.

The consistency of the relationship between N intake and urinary N yield (g/d) in our study, notwithstanding the limitations of the experimental model mentioned previously, indicate that increasing dietary CP through the addition of urea to a basal pasture diet could be used as an experimental model to test mitigation strategies aimed at reducing urinary N excretion. Future work should evaluate this method with larger numbers of cows and in pastures with differing basal dietary compositions to determine whether this experimental approach is valid under other nutritional situations.
